# State Space Modeling of Event Count Time Series

**DOI:** 10.3390/e25101372

**Published:** 2023-09-23

**Authors:** Sidratul Moontaha, Bert Arnrich, Andreas Galka

**Affiliations:** 1 Digital Health—Connected Healthcare, Hasso Plattner Institute, University of Potsdam, 14482 Potsdam, Germany; 2 Bundeswehr Technical Centre for Ships and Naval Weapons, Maritime Technology and Research (WTD 71), 24340 Eckernförde, Germany

**Keywords:** n onlinear state space model, iterated extended Kalman filter, Bayesian filtering, count time series, singular value decomposition

## Abstract

This paper proposes a class of algorithms for analyzing event count time series, based on state space modeling and Kalman filtering. While the dynamics of the state space model is kept Gaussian and linear, a nonlinear observation function is chosen. In order to estimate the states, an iterated extended Kalman filter is employed. Positive definiteness of covariance matrices is preserved by a square-root filtering approach, based on singular value decomposition. Non-negativity of the count data is ensured, either by an exponential observation function, or by a newly introduced “affinely distorted hyperbolic” observation function. The resulting algorithm is applied to time series of the daily number of seizures of drug-resistant epilepsy patients. This number may depend on dosages of simultaneously administered anti-epileptic drugs, their superposition effects, delay effects, and unknown factors, making the objective analysis of seizure counts time series arduous. For the purpose of validation, a simulation study is performed. The results of the time series analysis by state space modeling, using the dosages of the anti-epileptic drugs as external control inputs, provide a decision on the effect of the drugs in a particular patient, with respect to reducing or increasing the number of seizures.

## 1. Introduction

Temporal data sequences resulting from counting discrete events over a given time interval represent a particular variant of time series called discrete-valued or event count time series. These count data arise in various fields, such as physics, epidemiology, finance, econometrics, or medicine. Much of the existing framework on time series analysis relies on the assumptions of Gaussianity and linearity. The non-negativity and integrity constraints inherent in count time series have led to the development of alternative modeling approaches that instead employ probability distributions of Poisson type. As prominent examples we mention generalized linear models (GLMs) [1], dynamic generalized linear models [2,3], integer-valued autoregressive (INAR) models [4], and integer-valued generalized autoregressive conditional heteroscedasticity (INGARCH) models [5,6,7].

However, in this paper we aim at modeling count time series within the linear Gaussian regime as long as possible, while introducing non-negativity and integrity only at the last stage of modeling, namely, at the stage of modeling the observation process. The suitable framework for this agenda is given by classical state space modeling [8]. In state space modeling, the dynamical process underlying the data is explicitly separated from the observation process, such that the former can be kept Gaussian and linear, which considerably simplifies state estimation and model identification. Further advantages of the state space approach are given by the possibility to discriminate between dynamical noise and observation noise, and by the option of straightforward generalization to the multivariate case. It is also possible to include explanatory factors and external control inputs into state space models, and to incorporate conditional heteroscedasticity [9], with little additional effort.

Since the dynamics is kept Gaussian and linear, temporal correlations in the given data can be modeled by standard models from linear time series analysis, such as linear autoregressive moving average (ARMA) models [10]; ARMA models can be rewritten as (components of) linear state space models. Finally, non-negativity is modeled by employing a nonlinear observation function, while integrity is interpreted as the effect of a suitable additive observation noise term, which performs a kind of quantization of the non-integer output of the nonlinear observation function.

In linear state space modeling, tasks such as prediction, filtering, and smoothing may be performed by algorithms based on the linear Kalman filter (KF) [8,11]. However, in the case of a nonlinear observation function, generalizations of the linear KF need to be employed. Nowadays, a variety of algorithms is available, such as the extended KF (EKF), the iterated extended KF (IEKF), the unscented KF (UKF), and particle filtering [11,12].

The EKF is based on applying a linear KF by forming the local derivative of the nonlinear observation function (and the dynamical process, if it is also nonlinear) at the current estimate of the predicted state. The IEKF extends the EKF by an additional iteration that aims at finding consistent estimates for predicted and filtered states. It has recently been shown that the IEKF iteration can be interpreted as an application of Gauss–Newton optimization [12]. The UKF generalizes the EKF by propagating an ensemble of deterministically chosen points, thus improving Gaussian approximation and eliminating the explicit calculation of the Jacobian matrices. In principle, in Kalman filtering the error covariance matrices of the state estimates should be bounded and should converge to a steady solution, irrespective of the error in the initial state estimate and the corresponding initial error covariance matrix. However, the opposite phenomenon called *theoretical divergence* of classical KF is also well known. In order to keep the covariance matrix positive definite and improve the numerical behavior of the KF, square-root variants of the linear KF and its nonlinear generalizations have been introduced [13].

The mentioned algorithms have played a prominent role in applications in biomedical research and adjacent fields, such as public health [14]. Examples of corresponding count time series include epidemiological data (such as the famous U.S. poliomyelitis incidence time series, consisting of monthly counts starting in 1970) [15], sleep stage sequences, erythrocyte counts, infectious disease data [16], and epileptic seizure counts [17,18]. While most epilepsies respond well to anti-epileptic treatment, modeling the effects of anti-epileptic drugs (AEDs) on seizure frequency is essential for patients with difficult-to-treat or treatment-resistant epilepsies [19,20].

Several quantitative approaches to the analysis of seizure count time series have been proposed, which suffer from significant deficiencies. The *mixed-effects models* employed by Tharayil et al. cannot assess the effects of the changes in AEDs [21]. The epilepsy seizure management tool (*EpiSAT*) proposed by Chiang et al. does not account for observation errors caused by missed seizures or misinterpreted non-seizure events [22]. The Bayesian negative binomial dynamic linear model, recently proposed by Wang et al., cannot model the interaction effect between AEDs [23].

Application of state space modeling and Kalman filtering to seizure count time series has the potential to solve these deficiencies by quantifying the effect of an AED in the presence of other AEDs, describing delays in the effect of each AED, and modeling interaction effects between several AEDs. Moreover, the state space approach allows for the presence of temporal correlations in the seizure count time series that are unrelated to the current AED medication, but may result from other unknown influences on the probability of seizures. Furthermore, the state space approach is robust with respect to observation errors, such as missed seizures, events misclassified as seizures, outliers, missing data, or other observer-related errors. Therefore, the primary objective of this contribution is to explore and develop state space modeling algorithms tailored explicitly for event count time series, with a particular focus on the modeling of seizure count time series, as an illustrative example. To the best of our knowledge, apart from our previous work [24,25,26], this approach is novel on its own.

This paper is organized as follows. In Section 2, “Materials and Methods”, we discuss the main algorithms for Kalman filtering and for parameter estimation; we also describe the simulated data and the real data from a patient. In Section 3, “Results”, we show the performance of the proposed algorithm, provide some comparison with respect to the numerical problems arising in previous algorithms, and show results from application to both simulated and real data. Section 4, “Discussion”, concludes the paper. Additional information on the proposed state space models and Kalman filtering algorithms is provided in Appendices Appendix A and Appendix B.

## 2. Materials and Methods

### 2.1. Independent Components Linear State Space (IC-LSS) Models

The independent components linear state space (IC-LSS) model is a distinct category of the linear state space (LSS) models proposed by Galka et al. [27]. Let the data vector observed at time *t* be denoted by $y_{t}$, where t=1,…,T denotes discrete time, and let the dimension of $y_{t}$ be denoted by *n*. The examples for analysis of actual data that are shown below are for scalar data only, i.e., n=1, but we choose to keep the presentation of the methodology more general.

In linear state space (LSS) modeling, the observed data are linked to an unobserved *m*-dimensional state vector, $x_{t}$, as described by an observation equation

(1) $$y_{t}=Cx_{t}+\epsilon_{t},\;\begin{array}{c} \epsilon_{t}∼N\left(0,R\right) \end{array}$$

where C and $\epsilon_{t}$ represent the *observation matrix* and the *observation noise*, respectively. The observation noise is a white Gaussian noise with zero mean and *observation noise covariance matrix* R. By including this noise, the model acknowledges the limitations and uncertainties of real-world observations, and the information loss about the true state of the system. Within LSS models, the temporal evolution of the state vector, $x_{t}$, is described by a discrete-time dynamical equation

(2) $$x_{t}=Ax_{t-1}+\eta_{t},\;\begin{array}{c} \eta_{t}∼N\left(0,Q\right) \end{array}$$

where A and $\eta_{t}$ represent the *state transition matrix* and the *dynamical noise*, respectively. Also, the dynamical noise is a white Gaussian noise with zero mean and *dynamical noise covariance matrix* Q. An additional control input term, depending on a known external control input, $u_{t}$, may be added to the dynamical equation:

(3) $$x_{t}=Ax_{t-1}+B_{u}u_{t}+\eta_{t}$$

where $B_{u}$ represents the *control gain matrix*. The respective dimensions of the matrices and vectors are given in Table 1. The IC-LSS model is a specific subset of the LSS model family that characterizes data as a combination of independent source processes through a weighted sum and chooses specific structures of parameter matrices of the general state space model. The model depends on a set of parameters matrices, collected in the set $\Theta =\{C,A,R,Q,B_{u}\}$. Both the state transition matrix, A, and the dynamical noise covariance matrix, Q, are constructed as block-diagonal matrices with identical sets of block dimensions, as described in Appendix A.

Our modeling approach assumes that the impact of each control input, i.e., each component of the vector $u_{t}$, is independent of the other components, and that the corresponding processes can be modeled as deterministic first-order autoregressive models, to be denoted by AR(1). These AR(1) models are made deterministic by setting the corresponding elements of Q to zero. To account for temporally correlated fluctuations in the data caused by factors other than the control input, a stochastic process is also included. This stochastic process is modeled by an autoregressive moving average model with orders *p* and p−1, to be denoted as ARMA(p,p−1). In the current implementation, we usually choose p=2, which represents a trade-off between the stochasticity and stability of the model. AR(1) and ARMA(2,1) models can easily be incorporated into IC-LSS models (for technical details, see Appendix A).

### 2.2. Nonlinear State Space (NLSS) Models

Count time series do not follow Gaussian probability distributions, therefore the class of linear Gaussian state space models is not well suited for modeling such time series; rather it would be beneficial to employ appropriate nonlinear state space (NLSS) models. In this paper, we keep the dynamical equation linear, as in Equation (3), while defining a nonlinear observation equation by

(4) $$y_{t}=f\left(x_{t}\right)+\epsilon_{t},\;\begin{array}{c} \epsilon_{t}∼N\left(0,R\right) \end{array}$$

where we have assumed that the observation noise, $\epsilon_{t}$, can be kept Gaussian; f(.) represents a nonlinear observation function. We employ two different nonlinear functions, namely

(5) $$\begin{array}{c} f_{1}\left(x\right)=\exp\left(Cx\right) \end{array}$$

and

(6) $$\begin{array}{c} f_{2}\left(x\right)=\frac{Cx}{2}+\sqrt{\frac{{\left(Cx\right)}^{2}}{4}+k} \end{array}$$

In the case of multivariate data, n>1, these functions are to be applied component-wise.

**Figure 1:**
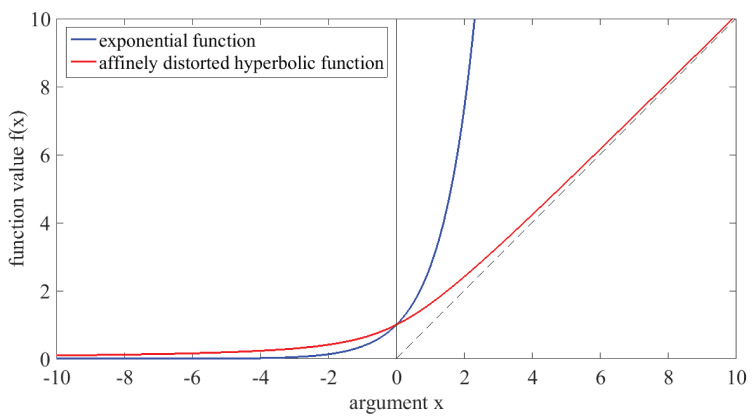
Nonlinear observation functions $f_{1}\left(x\right)$ (blue) and $f_{2}\left(x\right)$ (red), with C=1 and k=1; the dashed vertical line on the right side represents the linear function f(x)=x.

The first of these observation functions, simply an exponential function, has been chosen with the intention of achieving non-negativity of the observed data, as it may also be achieved in Poisson regression. The disadvantage of this choice for the observation function is the fact that it diverges exponentially for large positive arguments. In previous work, we have occasionally encountered numerical breakdown of Kalman filtering algorithms due to the resulting extremely large values [25,26]; details will be provided below in Section 3.1. For this reason, we propose a different nonlinear observation function, to be called the “affinely distorted hyperbolic” function, as given in Equation (6); while for negative arguments it behaves like the exponential function, for positive arguments it converges to the linear function, rising with a slope of C, see Figure 1.

Recently, Weiß and coworkers [7,28] have introduced a nonlinear function called the “softplus” function, given by

$$f\left(x\right)=k\log\left(1+\exp\left(x/k\right)\right)$$

where *k* is a real positive parameter. This function could be employed instead of our “affinely distorted hyperbolic” function for the purpose of constraining observations to be non-negative, since it has similar behavior.

### 2.3. Iterated Extended Kalman Filter (IEKF)

In this paper, we are dealing with a two-fold estimation problem: estimation of states and estimation of parameters. The extended Kalman filter (EKF) has been used as a popular tool for estimating the states in nonlinear state space models. It was first proposed by Kalman and Bucy in 1961 [29]. The EKF results from extending the original Kalman filter developed for linear systems to nonlinear systems by linearizing the dynamical equation and the observation equation around the current state estimate. As an improved variant of EKF, the iterated extended Kalman filter (IEKF) has been proposed, in order to improve the accuracy and stability of the EKF [30]. The IEKF has been applied to various fields, including robotics, aerospace, and control systems.

For a given time series of length *T* and given initial state estimate $x_{0|0}$ and corresponding covariance matrix $P_{0|0}$, the forward temporal recursion begins at time t=1 with the predicted state estimate [25], which is computed by

(7) $$\begin{array}{c} x_{t|t-1}=Ax_{t-1|t-1}+B_{u}u_{t} \end{array}$$

The notation $x_{t_{1}|t_{0}}$ is used throughout the paper to indicate that an estimate at time $t_{1}$ is obtained by using all data available at time $t_{0}$. The corresponding predicted state covariance is computed by

(8) $$\begin{array}{c} P_{t|t-1}=AP_{t-1|t-1}A^{T}+Q \end{array}$$

At each time point, the IEKF iteration starts, after Equations (7) and (8) have been evaluated, with iteration index $i=1,\ldots ,i_{m}$. First, the derivative of the chosen nonlinear function is computed as

(9) $$H_{t}^{\left(i\right)}=\frac{\partial f}{\partial x}{|}_{x_{t|t}^{\left(i-1\right)}}$$

In the multivariate case, this derivative will be a matrix (Jacobian matrix). At each iteration, the prediction errors (also known as *innovations*), the innovation variance, the Kalman gain, and the filtered state estimates are computed, according to

(10) $$\begin{array}{cc} \nu_{t}^{\left(i\right)} & =y_{t}-f\left(x_{t|t}^{\left(i-1\right)}\right) \end{array}$$

(11) $$\begin{array}{cc} V_{t}^{\left(i\right)} & =H_{t}^{\left(i\right)}P_{t|t-1}{\left(H_{t}^{\left(i\right)}\right)}^{T}+R \end{array}$$

(12) $$\begin{array}{cc} K_{t}^{\left(i\right)} & =P_{t|t-1}{\left(H_{t}^{\left(i\right)}\right)}^{T}{\left(V_{t}^{\left(i\right)}\right)}^{-1} \end{array}$$

(13) $$\begin{array}{cc} x_{t|t}^{\left(i\right)} & =x_{t|t-1}+K_{t}^{\left(i\right)}\left(\nu_{t}^{\left(i\right)}-H_{t}^{\left(i\right)}\left(x_{t|t-1}-x_{t|t}^{\left(i-1\right)}\right)\right) \end{array}$$

The iteration ends when a stopping criterion is fulfilled. We use the stopping criterion of either the norm of the relative change of $x_{t|t}^{\left(i\right)}$ falling below $10^{-10}$, or the iteration index reaching a maximal value of $i_{m}=100$. After the iteration, the filtered state covariance is computed by

(14) $$P_{t|t}=\left(I_{m}-K_{t}^{\left(i\right)}H_{t}^{\left(i\right)}\right)P_{t|t-1}$$

where $I_{m}$ denotes the *m*-dimensional identity matrix.

The IEKF algorithm, as presented above, is summarized in Appendix B. In an earlier paper [25], we have presented results from actual application of the IEKF, for the case of an exponential observation function.

### 2.4. Singular Value Decomposition Iterated Extended Kalman Filter (SVD-IEKF)

At each time step, the covariance matrices $P_{t|t-1}$, $V_{t}$, and $P_{t|t}$ arising in the Kalman filter recursion have to be positive definite, or at least, positive semi-definite. However, it is well known that during the recursion due to numerical effects these covariance matrices may lose the property of positive definiteness. As a consequence, computation of the likelihood becomes unreliable; furthermore, the iteration of the IEKF may converge only with delays, or it may entirely fail to converge [25].

As a remedy, one may work with matrix square roots of the covariance matrices, instead of the covariance matrices themselves. There are, at least, two ways to define the square root of a matrix: by Cholesky decomposition (CD) and by singular value decomposition (SVD).

For a given real square matrix M with dimension m×m, the Cholesky decomposition is given by

(15) $$M=S_{M}^{T}S_{M}$$

where $S_{M}$ denotes a (m×m)-dimensional upper triangular matrix with non-negative diagonal elements. The Cholesky Decomposition is only possible for matrices that are positive (semi-)definite; for semi-definiteness, the decomposition may not be unique.

For a given real matrix, M, with dimension m×n, the SVD decomposition is given by

(16) $$M=U_{M}\Sigma_{M}W_{M}^{T}$$

where $U_{M}$ and $W_{M}$ denote two orthogonal matrices with dimensions m×m and n×n, respectively, and $\Sigma_{M}$ is a diagonal matrix with dimension m×n, which has non-negative real numbers, $\sigma_{i}$, on its diagonal, called the singular values of M [31]. The singular values are the positive square roots of the eigenvalues of $M^{T}M$ [13]. SVD can be applied to any matrix, without the condition of positive (semi-)definiteness.

The SVD of a positive (semi-)definite square matrix, M, e.g., a covariance matrix, can be formulated as

(17) $$M=W_{M}\Sigma_{M}^{2}W_{M}^{T}$$

such that we have $U_{M}=W_{M}$, and the definition of the matrix of singular values has been changed to $\Sigma_{M}^{2}$ instead of $\Sigma_{M}$; this is a reasonable change of definition, since the singular values are non-negative.

Square-root variants of the Kalman filter that employ CD were proposed already in the 1960s. However, SVD represents a matrix decomposition that offers superior numerical properties, compared with CD. Square-root variants of the Kalman filter that employ SVD were proposed in 1992 by Wang et al. [13], and in 2017 by Kulikova and Tsyganova [32]. In an earlier paper, we proposed a square-root variant of the IEKF that employs SVD [26], based on the algorithm of Kulikova and Tsyganova.

In the proposed nonlinear Kalman filter algorithm, the initial covariance matrix, $P_{0|0}$, and the noise covariance matrices, Q and R, are factorized by SVD as follows:

$$\begin{array}{cc} P_{0|0} & =W_{0|0}\Sigma_{0|0}^{2}W_{0|0}^{T}\\ Q & =W_{Q}\Sigma_{Q}^{2}W_{Q}^{T}\\ R & =W_{R}\Sigma_{R}^{2}W_{R}^{T} \end{array}$$

These factorizations are performed once outside of the forward recursion of the Kalman filter. The recursion then begins with the same update equation for the predicted state as before, Equation (7). However, now the corresponding predicted state covariance matrix is updated by performing a factorization step by applying SVD to a *pre-array*, as follows [26]:

(18) $$\begin{array}{cc} U_{1}\underset{\mathrm{singular}\;\mathrm{values}\;\mathrm{array}}{\underset{︸}{\left[\begin{array}{c} \Sigma_{P_{t|t-1}}\\ 0 \end{array}\right]}}W_{P_{t|t-1}}^{T}= & \underset{\mathrm{pre}-\mathrm{array}}{\underset{︸}{\left[\begin{array}{c} \Sigma_{P_{t-1|t-1}}W_{P_{t-1|t-1}}^{T}A^{T}\\ \Sigma_{Q}W_{Q}^{T} \end{array}\right]}} \end{array}$$

(19) $$\begin{array}{cc} P_{t|t-1}= & W_{P_{t|t-1}}\Sigma_{P_{t|t-1}}^{2}W_{P_{t|t-1}}^{T} \end{array}$$

where $U_{1}$ is an orthogonal matrix that can be discarded. Also, in the SVD-IEKF algorithm, at each time point an iteration is performed, with the equations for the Jacobi matrix and the innovations given by Equations (9) and (10), respectively. However, Equation (11) is replaced by an array factorization step:

(20) $$\begin{array}{cc} U_{2}\underset{\mathrm{singular}\;\mathrm{values}\;\mathrm{array}}{\underset{︸}{\left[\begin{array}{c} \Sigma_{V_{t}}^{\left(i\right)}\\ 0 \end{array}\right]}}{\left(W_{V_{t}}^{\left(i\right)}\right)}^{T}\; & =\underset{\mathrm{pre}-\mathrm{array}}{\underset{︸}{\left[\begin{array}{c} \Sigma_{R}W_{R}^{T}\\ \Sigma_{P_{t|t-1}}^{\left(i\right)}{\left(W_{P_{t|t-1}}^{\left(i\right)}\right)}^{T}{\left(H_{t}^{\left(i\right)}\right)}^{T} \end{array}\right]}} \end{array}$$

(21) $$\begin{array}{cc} V_{t}^{\left(i\right)} & =W_{V_{t}}^{\left(i\right)}{\left(\Sigma_{V_{t}}^{\left(i\right)}\right)}^{2}{\left(W_{V_{t}}^{\left(i\right)}\right)}^{T} \end{array}$$

where $U_{2}$ is an orthogonal matrix which can be discarded. As the iteration proceeds, the normalized innovation, the normalized gain, the optimal Kalman gain, and the filtered state estimate are computed as follows:

(22) $$\begin{array}{cc} {\hat{\nu }}_{t}^{\left(i\right)} & ={\left(W_{V_{t}}^{\left(i\right)}\right)}^{T}\nu_{t}^{\left(i\right)} \end{array}$$

(23) $$\begin{array}{cc} k_{t}^{\left(i\right)} & =P_{t|t-1}{\left(H_{t}^{\left(i\right)}\right)}^{T}W_{V_{t}}^{\left(i\right)} \end{array}$$

(24) $$\begin{array}{cc} K_{t}^{\left(i\right)} & =k_{t}^{\left(i\right)}{\left(\Sigma_{V_{t}}^{\left(i\right)}\right)}^{-2}{\left(W_{V_{t}}^{\left(i\right)}\right)}^{T} \end{array}$$

(25) $$\begin{array}{cc} x_{t|t}^{\left(i+1\right)} & =x_{t|t-1}+k_{t}^{\left(i\right)}{\left(\Sigma_{V_{t}}^{\left(i\right)}\right)}^{-2}\left({\hat{\nu }}_{t}^{\left(i\right)}-{\left(W_{V_{t}}^{\left(i\right)}\right)}^{T}H_{t}^{\left(i\right)}\;\left(x_{t|t-1}-x_{t|t}^{\left(i\right)}\right)\right). \end{array}$$

After reaching the stopping criterion of the iteration, the filtered state covariance matrix, $P_{t|t}$, is computed by a third array factorization step:

(26) $$\begin{array}{cc} U_{3}\underset{\mathrm{singular}\;\mathrm{values}\;\mathrm{array}}{\underset{︸}{\left[\begin{array}{c} \Sigma_{P_{t|t}}\\ 0 \end{array}\right]}}W_{P_{t|t}}^{T}\; & =\underset{\mathrm{pre}-\mathrm{array}}{\underset{︸}{\left[\begin{array}{c} \Sigma_{P_{t|t-1}}W_{P_{t|t-1}}^{T}{\left(I_{m}-K_{t}^{\left(i\right)}H_{t}^{\left(i\right)}\right)}^{T}\\ \Sigma_{R}W_{R}^{T}{\left(K_{t}^{\left(i\right)}\right)}^{T} \end{array}\right]}} \end{array}$$

(27) $$\begin{array}{cc} P_{t|t} & =W_{P_{t|t}}\Sigma_{P_{t|t}}^{2}W_{P_{t|t}}^{T} \end{array}$$

where $U_{3}$ denotes another orthogonal matrix that can be discarded, and *i* denotes the index at which the iteration had stopped. The SVD-IEKF algorithm, as presented above, is summarized in Appendix B.

### 2.5. Non-Dynamic Regression Model: Gaussian Case

For the purpose of comparison, we also employ two types of non-dynamic regression models. The first of these models represents the classical linear Gaussian case; it is defined as follows:

(28) $$y_{t}={\tilde{B}}_{u}u_{t}+n_{t}$$

where ${\tilde{B}}_{u}$ denotes a matrix of regression coefficients, and $n_{t}$ denotes a time series of regression residuals, assumed to have a Gaussian distribution with zero mean and covariance matrix $\Sigma_{n}$. ${\tilde{B}}_{u}$ can be estimated by ordinary least squares; the covariance matrix, $\Sigma_{n}$, can then be computed as

(29) $$\Sigma_{n}=\frac{1}{T}\sum_{t=1}^{T}\left(y_{t}-{\tilde{B}}_{u}u_{t}\right){\left(y_{t}-{\tilde{B}}_{u}u_{t}\right)}^{T}$$

Note that the regression model of Equation (28) implicitly assumes that the effects of the different components of the control vector, $u_{t}$, are uncorrelated.

From the regression model of Equation (28), a logarithmic likelihood can be computed by

(30) $$\log L\left({\tilde{B}}_{u}\right)=-\frac{T}{2}\left(\log|\Sigma_{n}|+n\log2\pi +n\right)$$

### 2.6. Non-Dynamic Regression Model: Poisson Case

The second non-dynamic regression model is an example of a generalized linear model, representing the Poisson case; it is defined as follows:

(31) $$\log y_{t}={\tilde{B}}_{u}u_{t}+n_{t}$$

where ${\tilde{B}}_{u}$ denotes another matrix of regression coefficients, and $n_{t}$ denotes another time series of regression residuals; in this case, $y_{t}$ is assumed to have a Poisson distribution. Note that, for simplicity, we formulate this model only for the case of scalar data.

From the regression model of Equation (31), a logarithmic likelihood can be computed by

(32) $$\log L\left({\tilde{B}}_{u}\right)=\sum_{t=1}^{T}\left(y_{t}{\tilde{B}}_{u}u_{t}-\exp\left({\tilde{B}}_{u}u_{t}\right)-\log\left(y_{t}!\right)\right).$$

### 2.7. Parameter Estimation and Ensembles of Models

As mentioned earlier, when fitting a state space model to given data we have to solve a two-fold estimation problem: estimation of states and estimation of parameters. In this paper, estimation of the model parameters, denoted by Θ in Section 2.1, is performed by numerical maximization of the logarithmic innovation likelihood, denoted by logL(Θ), employing the Broyden–Fletcher–Goldfarb–Shanno (BFGS) quasi-Newton and Nelder–Mead simplex algorithms [33]. Apart from filtered state estimates, the forward recursion of the Kalman filter also provides the corresponding contributions to the logarithmic innovation likelihood, which may then be summed up:

(33) $$\log L\left(\Theta \right)=-\frac{1}{2}\sum_{t=1}^{T}\left(\log|V_{t}|+\nu_{t}^{T}V_{t}^{-1}\nu_{t}\right)-\frac{nT}{2}\log2\pi$$

In this expression, the effect of the initial state has been ignored. If the innovations, $\nu_{t}$, have been obtained by the IEKF or SVD-IEKF algorithms, their values correspond to the final values resulting from the iteration performed at time *t*, until the stopping criterion is fulfilled.

In order to avoid parameter redundancy with respect to the control gain matrix, $B_{u}$, the observation matrix, C, has not been included in the set of parameters to be estimated by optimization; instead constant values of 1.0 are employed, except for the ARMA(2,1) component, for which the control gain parameters are fixed at zero.

For given data, comparison of the performance of state space models, as discussed above in Section 2.2, Section 2.3 and Section 2.4, with the performance of non-dynamic regression models, as discussed above in Section 2.5 and Section 2.6, can be performed by comparison of the corresponding values of an information criterion, such as the (corrected) Akaike information criterion (AICc) [27]. The AICc can be computed from the logarithmic likelihood according to

(34) $$\mathrm{AICc}=-2\log L\left(\Theta \right)+\frac{2N_{\mathrm{par}}T}{T-N_{\mathrm{par}}-1}$$

where $N_{\mathrm{par}}$ denotes the number of data-adaptive parameters of the corresponding model; in the case of state space models, this would be the total number of parameters in Θ; if the nonlinear observation function, f(.), according to Equation (6), is chosen, also the parameter *k* becomes part of Θ.

The second term in Equation (34) represents a penalty term for the complexity of the model; through this term, the values of AICc for different models can be directly compared, while the corresponding values of the logarithmic likelihood cannot, since they would be biased in favor of the more complex model. The task of parameter estimation is then given by finding parameters that minimize the AICc. Due to the complicated dependence of the AICc on Θ, including the possible existence of numerous local minima, this task can only be approached by numerical optimization. The chosen algorithm for numerical optimization may converge to one of these local minima, instead of the global minimum, or to other minima with smaller AICc values.

This issue can be resolved by employing an ensemble of models. Each model in the ensemble is initialized with randomly selected initial parameter values and optimized using the same minimum AICc approach until convergence. Finally, the model with the smallest AICc value is retained. However, although the probability of actually finding the global minimum will be improved by this ensemble approach, no assurance of finding the global minimum is provided.

### 2.8. Simulated Data

**Figure 2:**
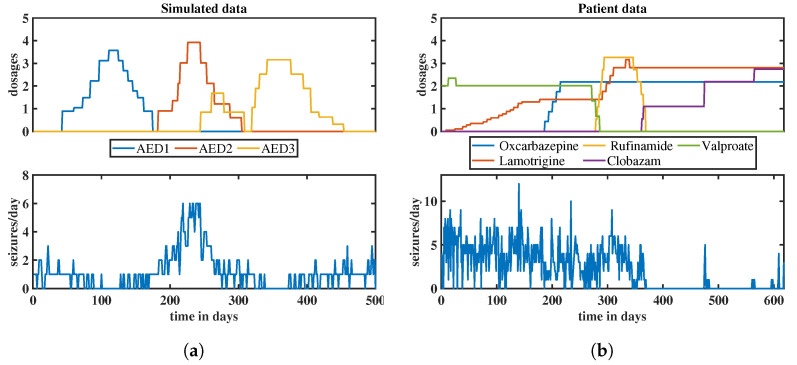
Time-dependent dosages of anti-epileptic drugs (upper panels) and corresponding count time series of daily epileptic seizures (lower panels) for a simulation (**a**) and for a real patient (**b**); in the upper panels, the anti-epileptic drugs are discriminated by colors (see legends below the panels, which also give the names of the drugs).

To validate the performance and effectiveness of our proposed NLSS modeling approach, we conduct simulations, before applying it to the patient data. These simulations serve to demonstrate the algorithm’s capability in accurately capturing the dynamics of event count time series. We assume that three anti-epileptic drugs are given, named AED1, AED2 and AED3, and that, for the particular simulated “patient”, AED1 and AED3 reduce the daily number of seizures, while AED2 increases it. The (arbitrarily chosen) time-dependent dosages of the drugs during a time interval of 500 days are shown in the upper panel of Figure 2a, while the resulting time series of the daily numbers of seizures is shown in the lower panel of the figure.

The simulated time series is generated by employing Equations (1) and (3), utilizing the affinely distorted hyperbolic function $f_{2}\left(x_{t}\right)$, as discussed in Equation (6). In addition to the contributions of the three anti-epileptic drugs, a stochastic ARMA(2,1) process is added. The resulting time series is rounded to integer values, representing the simulated daily seizure counts. The daily seizure counts assume values from 0 to 6, which is a realistic interval. The MATLAB code for recreating the simulated data is provided in Appendix C.

### 2.9. Patient Data

We demonstrate the practical application of NLSS modeling through the analysis of a real-world data set obtained from a patient suffering from symptomatic epilepsy. The data set utilized in this study was collected from an electronic seizure diary called EPI-Vista (http://www.epivista.de, accessed on 20 September 2023), which has been in routine use at the North German Epilepsy Center for Children and Adolescents in Schwentinental-Raisdorf, Germany, since 2007. EPI-Vista is a freely available therapy management tool that records information about dosages of administered anti-epileptic drugs and about seizure events.

The time-dependent dosages of the drugs during the chosen time interval of 618 days are shown in the upper panel of Figure 2b, while the recorded time series of the daily numbers of seizures is shown in the lower panel of the figure. During the chosen time interval, five different anti-epileptic drugs were administered: oxcarbazepine, lamotrigine, rufinamide, clobazam, and valproate.

## 3. Results

### 3.1. Convergence Behavior of Iteration

**Figure 3:**
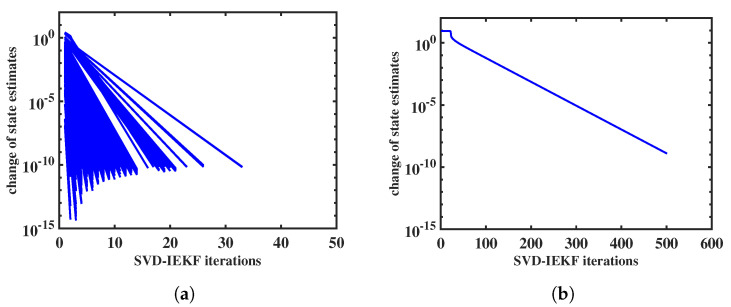
Norm of relative change of state estimate vs. iteration index for the application of the SVD-IEKF to a simulated count time series, using a model initialized with two different sets of random model parameters; in (**a**) iterations for all 500 time points are shown, while in (**b**) only the first iteration is shown, since the recursion of the Kalman filter was aborted afterwards due to numerical failure.

**Figure 4:**
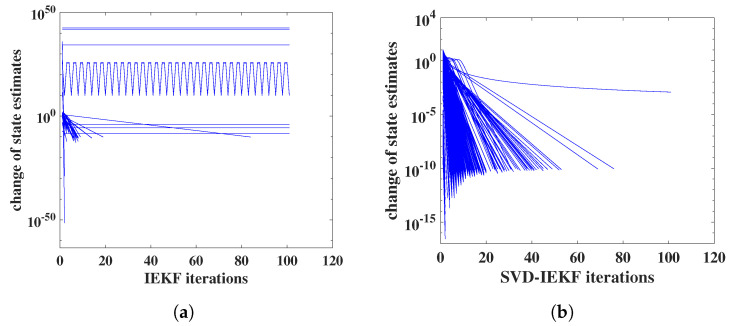
Norm of relative change of state estimate vs. iteration index for the application of the IEKF (**a**) or SVD-IEKF (**b**) to a simulated count time series, using a model initialized with a set of random model parameters; iterations for all 500 time points are shown.

We will briefly comment on the convergence behavior of the iteration of the SVD-IEKF. Within the recursion of the SVD-IEKF through the data, the iteration takes place at each time point. We may plot the norm of the relative change of the state estimate as a function of the iteration index, obtaining a set of curves; examples are shown in Figure 3 and Figure 4. These examples refer to the analysis of the simulated data, as described in Section 2.8. In Figure 3a, it can be seen that for all 500 time points the iteration converges according to a power law within, at most, 35 iterations, thereby confirming that the SVD-IEKF works properly.

However, within an ensemble of models, cases may also occur that show less favorable behavior. An example is shown in Figure 3b. In this case, we see convergence only for the first time point; the iteration loop stops after 500 iterations. State estimates obtained from this iteration were extremely large (in the order of $e^{100}$), and therefore the Kalman filter recursion was not continued, and no further iterations were performed. This problem can be resolved by replacing the exponential function with the affinely distorted hyperbolic function in the observation equation, Equation (1).

In Figure 4, we illustrate the effects of loss of positive definiteness of covariance matrices on the convergence behavior. Also, this example refers to the analysis of the simulated data. The standard IEKF was used, as described above in Section 2.3, and the affinely distorted hyperbolic function was employed.

In Figure 4a, it can be seen that for most time points the iteration completely fails to converge, instead norms of relative state changes stay approximately constant or oscillate; note the extremely large, or small, values on the vertical axis. In Figure 4b, the effect of switching from the IEKF to the SVD-IEKF is illustrated, for the same simulated data and the same set of model parameters: now, for almost all time points, good convergence within 60 iterations is obtained. For a single time point, convergence is slower and seems not to follow a power law.

Within an ensemble of 1000 random initial models, we find that for 18 models the IEKF encounters numerical problems resulting from covariance matrices losing positive definiteness or becoming singular, and another 11 models exhibit poor convergence behavior. For the SVD-IEKF, all models of the ensemble display good or satisfactory convergence behavior, without any numerical instability of the Kalman filter.

### 3.2. Results of Ensemble Approach: Simulated Data

**Figure 5:**
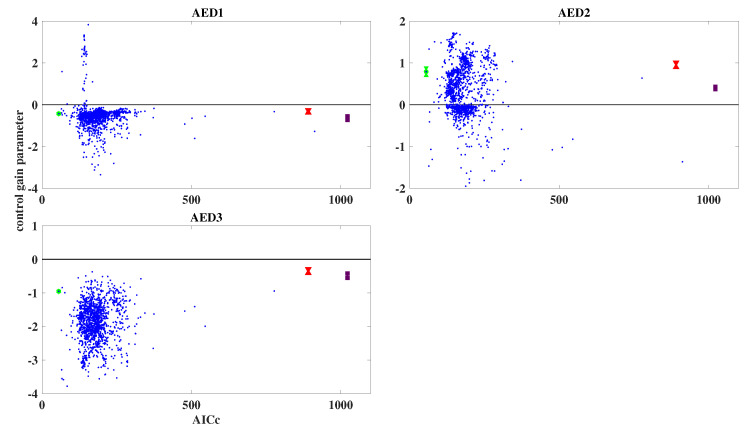
Estimated control gain parameters vs. AICc for simulated data, using an ensemble of 1000 randomly initialized models. The blue dots represent the results for the ensemble, with the model with the lowest AICc highlighted in green. The red (Gaussian case) and deep purple (Poisson case) dots represent the results obtained by the non-dynamic regression models. For the green, red, and deep purple dots, error bars are added, but they are mostly very small. The three panels refer to the three anti-epileptic drugs that were used in the simulation.

Following earlier work [24], we plot the control gain parameters, i.e., the diagonal elements of the control gain matrix, $B_{u}$, as defined in Equation (3), against the corresponding values of the AICc for all models of the ensemble; for each diagonal element, i.e., for each anti-epileptic drug, a separate plot is created. For the simulated data, the resulting plots are shown in Figure 5; blue dots represent the results from the 1000 models of the ensemble. The model with the lowest AICc value is highlighted in green. In addition, the results obtained by the two non-dynamic regression models are also shown, and are represented by red (Gaussian) and deep purple (Poisson); error bars are also shown, although they are mostly very small.

From Figure 5 it can be seen that most of the models of the ensemble achieve a lower AICc, compared with the non-dynamic regression models. For AED1 and AED2, the clouds of blue dots scatter over both positive and negative values of the control gain parameter; however, the model with the smallest control gain parameter is negative for AED1, and positive for AED2. For AED3, literally all models yield negative control gain parameters.

Since the simulation was designed such that AED1 and AED3 reduce the daily number of seizures, while AED2 increases it, the ensemble approach has succeeded in retrieving the correct result. As can be seen from Figure 5, in this case also the non-dynamic regression models reproduce the correct result.

In Table 2, the correct values of the observation parameters, which were used for creating the simulated data, and the estimated values of these parameters, obtained for the model with the lowest AICc value, are shown. The table also lists estimated errors for the estimated parameters; these errors can be estimated by computing the Hessian of the local likelihood at the optimal point. However, it is obvious that, at least for AED1 and AED3, these estimated errors are much too small to describe the actual deviation of the estimated values from the correct values; the probable reason for this is that in nonlinear filtering algorithms with an iteration at each time point, such as the IEKF and SVD-IEKF, the local likelihood often has a complicated shape with discontinuous behavior, such that numerically computed Hessians do not provide reliable estimates of the estimation errors.

### 3.3. Result of Ensemble Approach: Patient Data

**Figure 6:**
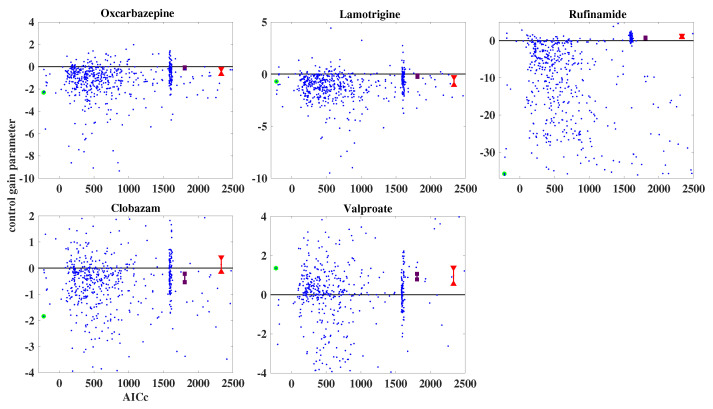
Estimated control gain parameters vs. AICc for patient data, using an ensemble of 700 randomly initialized models. The blue dots represent the results for the ensemble, with the model with the lowest AICc highlighted in green. The red (Gaussian case) and deep purple (Poisson case) dots represent the results obtained by the non-dynamic regression models. For the green, red, and deep purple dots, error bars are added. The five panels refer to the five anti-epileptic drugs that were administered during the chosen time interval of 618 days.

For the patient data, an ensemble of 700 randomly initialized models was employed. When using the SVD-IEKF and the affinely distorted hyperbolic function, optimization of all models proceeded without cases of numerical problems. The resulting plots of estimated control gain parameters against the corresponding values of the AICc for all models of the ensemble are shown in Figure 6; again, blue dots represent the results from the models of the ensemble, the model with the lowest AICc value is highlighted in green, and the results obtained by the two non-dynamic regression models are represented by red (Gaussian) and deep purple (Poisson).

From Figure 6 it can be seen that, again, most of the models of the ensemble achieve a lower AICc, compared with the non-dynamic regression models. The error bars of the red dots are somewhat larger now than in the case of the simulated data; for clobazam, the error interval includes the value of zero for the control gain parameter, such that it would become impossible to decide whether the effect of this anti-epileptic drug on the seizure count would be increasing, decreasing, or zero. Furthermore, based on the non-dynamic regression models, we would conclude that oxcarbazepine and lamotrigine would decrease the seizure count, while rufinamide and valproate would increase it.

On the other hand, according to the results of the analysis by the SVD-IEKF, we would conclude that all anti-epileptic drugs, except for valproate, would decrease the seizure count; consequently, for two anti-epileptic drugs, the conclusions would differ from the conclusions based on the non-dynamic regression models.

We add a comment on the results of the ensemble approach, both for simulated and patient data, as shown in Figure 5 and Figure 6. In this paper, we are dealing with time series of fairly short length, at most a few hundred values, since this is the typical situation for time series of a daily number of certain events, such as epileptic seizures. As a consequence of this scarcity of data, relative to the dimension of the space of model parameters, the AICc (i.e., the negative likelihood), as a function of these parameters, will display many local minima, and the task of finding the global minimum may not be well defined. For this reason, we consider it necessary to choose an ensemble approach. In the figures showing the results of the ensemble approach, the clouds of results that have a higher value of the AICc than the best model also contain some useful information.

As an example, consider again Figure 6. For each of the five anti-epileptic drugs displayed, it can be seen that the majority of the blue dots correspond to the same sign of the control gain parameter as the best model (i.e., the dot highlighted in green), thereby lending additional credibility to the resulting conclusions on the effects of these drugs. If, in such a case, the blue dots were distributed about equally over positive and negative values of the control gain parameter, we would be less inclined to regard the sign of this parameter for the best model as significant.

The underlying problem with respect to estimating model parameters from small data sets is unrelated to the particular choice of a state space model with linear dynamics and nonlinear observation; it has been observed quite similarly in an earlier study based on purely linear modeling [24]. The task to be addressed here is to draw the optimal conclusion, based on the scarce available data.

### 3.4. Innovation Whiteness Test

The aim of modeling a given time series by a parametric model, such as a state space model, is given by extracting temporal correlations as much as possible, such that the remaining prediction errors, i.e., the innovations, do not contain any residual correlations; this is equivalent to the innovations being white noise. In order to demonstrate that our modeling of the given data has been successful, with respect to this whiteness criterion, we will now show the autocorrelation function of the innovations of the best state space models from the ensembles.

**Figure 7:**
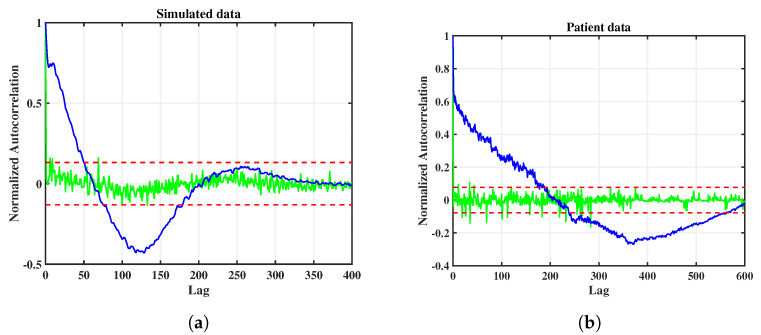
Autocorrelation functions of raw data (blue curves) and innovations after state space modeling (green curves) for simulated data (**a**) and patient data (**b**), for the best models of the respective ensembles. The red dashed lines correspond to one standard deviation of the innovations.

In Figure 7, the autocorrelation functions for the innovations of the simulated data (left panel) and the patient data (right panel) are shown (green curves); for comparison, the autocorrelation functions of the raw data are also shown (blue curves). The red dashed lines correspond to one standard deviation. Note that, at lag zero, autocorrelation functions always assume a value of 1.0. By comparison of the blue and the green curves it can be seen that, both for simulated data and for patient data, very good whitening has been achieved, with only a few outliers exceeding one standard deviation. This result confirms the validity, in a statistical sense, of the chosen approach of modeling the data by a state space model with a nonlinear observation function.

## 4. Discussion

In this paper, we propose an algorithm for analyzing event count time series by state space modeling and Kalman filtering. Within the larger field of time series analysis, the analysis of event count time series represents a special case, which is characterized by the need to model data that are both non-negative and integer, i.e., data given by natural numbers. The classical linear Gaussian state space model seems less suited for such data, since count data are described by Poisson distributions, rather than Gaussian distributions.

However, as we demonstrate, it is possible to keep the dynamics of the state space model Gaussian and linear, while introducing nonlinearity only at the last stage of modeling, namely, at the stage of modeling the observation process, thereby enforcing non-negativity and integrity of the observed data. The step from the non-integer output of the observation function to the integer data can then be roughly interpreted as addition of “quantization noise”. By this device, Poisson distributions need not be employed explicitly. Nevertheless, due to the choice of a nonlinear observation function, it is necessary to employ nonlinear Kalman filters.

The present paper summarizes and extends earlier work, according to a sequence of steps that can be described as follows:

- In an initial study, we had proposed to analyze event count time series by purely linear Gaussian state space modeling, using the standard linear Kalman filter [24].
- Then, as a generalization, we employed nonlinear state space modeling, such that a linear dynamical equation was combined with a nonlinear observation function; the exponential function was chosen. State space modeling was performed by the iterated extended Kalman filter (IEKF) [25].
- As a further step of improvement, the standard IEKF was replaced by the numerically superior singular value decomposition variant of the IEKF [26].
- In the present paper, we replaced the exponential function with the “affinely distorted hyperbolic” function; alternatively, the “softplus function” of Weiß and coworkers could also have been employed. We have not yet systematically compared both functions, but expect that they would display similar performance.

The classical linear Kalman filter consists of a recursion in the forward direction through time, which represents the optimal state estimator for linear Gaussian state space models [8]. As soon as nonlinearities are introduced into the dynamical equation or the observation equation, no closed-form optimal recursion exists, such that approximations and additional iterations have to be employed. The iterated extended Kalman filter (IEKF) represents a well-established example of such approximative nonlinear state estimators.

It is well known that, in the practical application of both the classical linear Kalman filter and its nonlinear generalizations, numerical problems may arise, which result from covariance matrices losing the property of positive definiteness. The usual remedy for such problems is given by expressing the recursion, not directly for the covariance matrices, but for square roots of these matrices, which is known as *square root filtering*. Matrix square roots may be defined either by Cholesky decomposition, or by singular value decomposition (SVD). Since the latter decomposition represents the more general and numerically more robust decomposition [34], we have chosen to employ it for our state estimation algorithm. The resulting algorithm is, to the best our knowledge, the first SVD variant of the IEKF that has been proposed [26].

When defining a state space model with a linear dynamical equation, the use of an exponential observation function represents a natural choice, in order to keep the data non-negative. However, the disadvantage of the exponential function is its exponential divergence for positive arguments, which may lead to numerical failure of the SVD-IEKF algorithm. For this reason, we propose a new nonlinear observation function that converges to zero for negative arguments, just like the exponential function, while converging to a linear function for positive arguments. This function can be derived by affinely distorting a (negative) hyperbolic function, such that the vertical axis becomes the desired linear function for positive arguments. As we have demonstrated in the present paper by analyzing both simulated and real data, employing the affinely distorted hyperbolic function within the SVD-IEKF algorithm finally removes the risk of numerical failure.

We emphasize that the proposed algorithm can be applied to any event count time series that one may wish to analyze under a non-negativity constraint; here, as an example, we have applied the algorithm to the analysis of time series of the daily number of seizures of drug-resistant epilepsy patients undergoing treatment with several, simultaneously administered anti-epileptic drugs. The time-dependent dosages of these drugs are inserted into the state space model as an external control input. The simultaneous presence of several drugs, their potential superposition effects, delay effects, and further unknown factors influencing the daily number of seizures make the objective analysis of seizure count time series arduous. As we have demonstrated, both in a simulation study and in the analysis of data from a real patient, state space modeling provides a powerful and flexible framework for analyzing the effects of anti-epileptic drugs in epilepsy patients. By comparison of an information criterion, such as the AICc, it can be proven that state space modeling provides a modeling of the data that is superior to modeling by conventional non-dynamic regression models.

There exist other model classes that take temporal correlations into account, such as the dynamic GLM, INAR, and INGARCH model classes. Our approach to modeling event count time series, as proposed in the present paper, represents an alternative to these model classes, but we do not intend to replace these classes, but rather provide an additional tool for the analysis of event count time series. According to a well-known proverb, “all models are wrong, but some models are useful”. Then, the justification of introducing a new class of models can come only from their usefulness in practical work, which needs to be explored by application to real data. Within the likelihood framework, the comparison of different model classes should be performed by quantifying their performance in predicting the data, preferably by using an information criterion, such as the AICc. It would be very interesting to perform a systematic study of the performance of our approach to modeling event count time series, in comparison with the already available model classes; this, however, is beyond the scope of the present paper and remains a task for future work.

With respect to the chosen field of application, namely, the analysis of time series of the daily number of seizures of drug-resistant epilepsy patients, in the future we intend to apply the presented algorithm to data from larger cohorts of patients. By discriminating between different types of seizures in the same patient, it is also intended to generalize the analysis to the case of vector data. Furthermore, the analysis should be generalized such that not only the effects of individual anti-epileptic drugs are modeled, but also the interaction effects between pairs, or groups, of drugs. Finally, in order to reduce the computational time consumption, work should be devoted to developing more efficient algorithms for fitting ensembles of nonlinear state space models by numerical maximum-likelihood procedures.

## Figures and Tables

**Table 1:** Quantities arising in state space models.

Notation	Meaning and/or Name	Dimension
$y_{t}$	data vector	*n*
$\epsilon_{t}$	observation noise vector	*n*
$x_{t}$	state vector	*m*
$\eta_{t}$	dynamical noise vector	*m*
$u_{t}$	external control vector	*u*
C	observation matrix	n×m
A	state transition matrix	m×m
R	observation noise covariance matrix	n×n
Q	dynamical noise covariance matrix	m×m
$B_{u}$	control gain matrix	m×u

**Table 2:** Correct and estimated values of the observation parameters for the simulation study; the third row gives estimated errors for the estimated values, obtained from the Hessian of the local likelihood.

Anti-Epileptic Drug	AED1	AED2	AED3
correct values	−0.40	0.95	−0.70
estimated values (best model)	−0.43	0.79	−0.96
estimated errors (best model)	$\pm 1.86\times 10^{-7}$	$\pm 4.57\times 10^{-2}$	$\pm 4.33\times 10^{-8}$

## Data Availability

The anonymized patient data set used in this paper is available for research purposes from the authors upon specific request.

## Abbreviations

     The following abbreviations are used in this paper:

AED	Anti-epileptic drug
IC-LSS	Independent components linear state space
NLSS	Nonlinear state space
AR	Autoregressive
ARMA	Autoregressive moving average
IEKF	Iterated extended Kalman filter
SVD	Singular value decomposition
CD	Cholesky decomposition
BFGS	Broyden–Fletcher–Goldfarb–Shanno
AICc	corrected Akaike information criterion

## Appendix A. Block Diagonal Structure of A and Q

The IC-LSS model discussed in Section 2.1 is based on a block-diagonal structure [35] of the state transition matrix, A, and the dynamical noise covariance matrix, Q, as follows [27]:

(A1) $$\begin{array}{c} A=\left(\begin{array}{ccccc} A_{1} & 0 & 0 & \cdots & 0\\ 0 & A_{2} & 0 & \cdots & 0\\ \vdots & \vdots & \vdots & \ddots & 0\\ 0 & 0 & 0 & \cdots & A_{J} \end{array}\right), \end{array}\;\begin{array}{c} Q=\left(\begin{array}{ccccc} Q_{1} & 0 & 0 & \cdots & 0\\ 0 & Q_{2} & 0 & \cdots & 0\\ \vdots & \vdots & \vdots & \ddots & 0\\ 0 & 0 & 0 & \cdots & Q_{J} \end{array}\right) \end{array}$$

where *J* denotes the number of independent components and also represents the number of blocks. If we assume that the *j*th block represents an autoregressive moving average (ARMA) model with model orders (p,p−1), consisting of an autoregressive part with parameters $a_{\tau }^{\left(j\right)}$, τ=1,…,p, and a moving average part with parameters $b_{\tau }^{\left(j\right)}$, τ=0,…,p−1, the block matrix, $A_{j}$, has left companion form, given by [27]   

(A2) $$\begin{array}{c} A_{j}=\left(\begin{array}{ccccc} a_{1}^{\left(j\right)} & 1 & 0 & \cdots & 0\\ a_{2}^{\left(j\right)} & 0 & 1 & \cdots & 0\\ \vdots & \vdots & \vdots & \ddots & 0\\ a_{p-1}^{\left(j\right)} & 0 & 0 & \cdots & 1\\ a_{p}^{\left(j\right)} & 0 & 0 & \cdots & 0 \end{array}\right) \end{array}$$

and the block matrix, $Q_{j}$, has outer product form, given by [27]

(A3) $$\begin{array}{c} Q_{j}=\left(\begin{array}{cccc} {\left(b_{0}^{\left(j\right)}\right)}^{2} & b_{0}^{\left(j\right)}b_{1}^{\left(j\right)} & \cdots & b_{0}^{\left(j\right)}b_{q}^{\left(j\right)}\\ b_{1}^{\left(j\right)}b_{0}^{\left(j\right)} & {\left(b_{1}^{\left(j\right)}\right)}^{2} & \cdots & b_{1}^{\left(j\right)}b_{q}^{\left(j\right)}\\ \vdots & \vdots & \vdots & \ddots \\ b_{p-2}^{\left(j\right)}b_{0}^{\left(j\right)} & b_{p-2}^{\left(j\right)}b_{1}^{\left(j\right)} & \cdots & b_{p-2}^{\left(j\right)}b_{q}^{\left(j\right)}\\ b_{p-1}^{\left(j\right)}b_{0}^{\left(j\right)} & b_{p-1}^{\left(j\right)}b_{1}^{\left(j\right)} & \cdots & {\left(b_{p-1}^{\left(j\right)}\right)}^{2} \end{array}\right) \end{array}$$

We usually employ the scaling convention $b_{0}^{\left(j\right)}=1$ for all blocks.

## Appendix B. Iterated Extended Kalman Filter (IEKF) Algorithms

**Algorithm** **A1** Iterated Extended Kalman Filter (IEKF)
**Require:** Initial state estimate: $x_{0|0}$ **Require:** Initial covariance matrix: $P_{0|0}$ 1: **for** t=1 to *T* **do** 2: **Prediction Step:** 3: Compute the predicted state estimate: $x_{t|t-1}=Ax_{t-1|t-1}+B_{u}u_{t}$ 4: Compute the predicted state covariance matrix: $P_{t|t-1}=AP_{t-1|t-1}A^{T}+Q$ 5: **Update Step:** 6: Initialize iteration: i=1, $x_{t|t}^{\left(0\right)}=x_{t|t-1}$ 7: **repeat** 8: Compute the Jacobian matrix of the observation function: $H_{t}^{\left(i\right)}=\frac{\partial f}{\partial x}{|}_{x_{t|t}^{\left(i-1\right)}}$ 9: Compute the innovation: $\nu_{t}^{\left(i\right)}=y_{t}-f\left(x_{t|t}^{\left(i-1\right)}\right)$ 10: Compute the innovation covariance matrix: $V_{t}^{\left(i\right)}=H_{t}^{\left(i\right)}P_{t|t-1}{\left(H_{t}^{\left(i\right)}\right)}^{T}+R$ 11: Compute the Kalman gain: $K_{t}^{\left(i\right)}=P_{t|t-1}{\left(H_{t}^{\left(i\right)}\right)}^{T}{\left(V_{t}^{\left(i\right)}\right)}^{-1}$ 12: Update the state estimate: $x_{t|t}^{\left(i\right)}=x_{t|t-1}+K_{t}^{\left(i\right)}\left(\nu_{t}^{\left(i\right)}-H_{t}^{\left(i\right)}\left(x_{t|t-1}-x_{t|t}^{\left(i-1\right)}\right)\right)$ 13: Increment iteration: i=i+1 14: **until** Stopping criterion met or maximum number of iterations ($i_{m}$) reached 15: Compute the filtered state covariance matrix: $P_{t|t}=\left(I_{m}-K_{t}^{\left(i\right)}H_{t}^{\left(i\right)}\right)P_{t|t-1}$ 16: **end for**

**Algorithm** **A2** Singular Value Decomposition Iterated Extended Kalman Filter (SVD-IEKF)
**Require:** Initial state estimate: $x_{0|0}$ **Require:** Initial covariance matrix: $P_{0|0}=W_{0|0}\Sigma_{0|0}^{2}W_{0|0}^{T}$ **Require:** Factorized dynamic noise covariance matrix: $Q=W_{Q}\Sigma_{Q}^{2}W_{Q}^{T}$ **Require:** Factorized observation noise covariance matrix: $R=W_{R}\Sigma_{R}^{2}W_{R}^{T}$ 1: **for** t=1 to *T* **do** 2: **Prediction Step:** 3: Compute the predicted state estimate: $x_{t|t-1}=Ax_{t-1|t-1}+B_{u}u_{t}$ 4: Compute the predicted state covariance matrix: $U_{1}\left[\begin{array}{c} \underset{0}{\Sigma_{P_{t|t-1}}} \end{array}\right]W_{P_{t|t-1}}^{T}=\left[\begin{array}{c} \underset{\Sigma_{Q}W_{Q}^{T}}{\Sigma_{P_{t-1|t-1}}W_{P_{t-1|t-1}}^{T}A^{T}} \end{array}\right]$ $P_{t|t-1}=W_{P_{t|t-1}}\Sigma_{P_{t|t-1}}^{2}W_{P_{t|t-1}}^{T}$ 5: **Update Step:** 6: Initialize iteration: i=1, $x_{t|t}^{\left(0\right)}=x_{t|t-1}$ 7: **repeat** 8: Compute the Jacobian matrix of the observation function: $H_{t}^{\left(i\right)}=\frac{\partial f}{\partial x}{|}_{x_{t|t}^{\left(i-1\right)}}$ 9: Compute the innovation: $\nu_{t}^{\left(i\right)}=y_{t}-f\left(x_{t|t}^{\left(i-1\right)}\right)$ 10: Compute the innovation covariance matrix: $U_{2}\left[\begin{array}{c} \underset{0}{\Sigma_{V_{t}}^{\left(i\right)}} \end{array}\right]{\left(W_{V_{t}}^{\left(i\right)}\right)}^{T}=\left[\begin{array}{c} \underset{\Sigma_{P_{t|t-1}}^{\left(i\right)}{\left(W_{P_{t|t-1}}^{\left(i\right)}\right)}^{T}{\left(H_{t}^{\left(i\right)}\right)}^{T}}{\Sigma_{R}W_{R}^{T}} \end{array}\right]$ $V_{t}^{\left(i\right)}=W_{V_{t}}^{\left(i\right)}{\left(\Sigma_{V_{t}}^{\left(i\right)}\right)}^{2}{\left(W_{V_{t}}^{\left(i\right)}\right)}^{T}$ 11: Compute normalized innovation: ${\hat{\nu }}_{t}^{\left(i\right)}={\left(W_{V_{t}}^{\left(i\right)}\right)}^{T}\nu_{t}^{\left(i\right)}$ 12: Compute normalized gain: $k_{t}^{\left(i\right)}=P_{t|t-1}{\left(H_{t}^{\left(i\right)}\right)}^{T}W_{V_{t}}^{\left(i\right)}$ 13: Compute the optimal Kalman gain: $K_{t}^{\left(i\right)}=k_{t}^{\left(i\right)}{\left(\Sigma_{V_{t}}^{\left(i\right)}\right)}^{-2}{\left(W_{V_{t}}^{\left(i\right)}\right)}^{T}$ 14: Update the state estimate: $x_{t|t}^{\left(i+1\right)}=x_{t|t-1}+k_{t}^{\left(i\right)}{\left(\Sigma_{V_{t}}^{\left(i\right)}\right)}^{-2}({\hat{\nu }}_{t}^{\left(i\right)}-{\left(W_{V_{t}}^{\left(i\right)}\right)}^{T}H_{t}^{\left(i\right)}\;\left(x_{t|t-1}-x_{t|t}^{\left(i\right)}\right)$ 15: Increment iteration: i=i+1 16: **until** Stopping criterion met or maximum number of iterations ($i_{m}$) reached 17: Compute the filtered state covariance matrix: $U_{3}\left[\begin{array}{c} \underset{0}{\Sigma_{P_{t|t}}} \end{array}\right]W_{P_{t|t}}^{T}=\left[\begin{array}{c} \underset{\Sigma_{R}W_{R}^{T}{\left(K_{t}^{\left(i\right)}\right)}^{T}}{\Sigma_{P_{t|t-1}}W_{P_{t|t-1}}^{T}{\left(I_{m}-K_{t}^{\left(i\right)}H_{t}^{\left(i\right)}\right)}^{T}} \end{array}\right]$ $P_{t|t}=W_{P_{t|t}}\Sigma_{P_{t|t}}^{2}W_{P_{t|t}}^{T}$ 18: **end for**

## Appendix C. Matlab Code for Generating Simulated Data

**Listing** **A1.** MATLAB code for generating simulated data
